# Interspecies encounters with endemic health conditions: co-producing BVD and lameness with cows and sheep in the north of England

**DOI:** 10.1111/soru.12458

**Published:** 2023-10-05

**Authors:** L Holloway, N Mahon, B Clark, A Proctor

**Keywords:** Farming, cows and sheep, care, nonhuman agency, subjectivity, endemic disease, UK

## Abstract

This paper focuses on the relationships between people and farmed nonhuman animals, and between these animals and the farmed environments they encounter, in the enactment of interspecies endemic disease situations. It examines how the nonhuman embodied capacities, agency and subjectivities of cows and sheep on farms in the north of England make a difference to how the endemic conditions of lameness and bovine viral diarrhoea (BVD) are encountered and responded to by farmers and advisers. The paper draws on empirical research with farmers and their advisers, and explores three key, inter-related, themes: first, the importance of intersubjective relationships between people and animals on farms; second, the nonhuman components of the ‘disease situations’ associated with endemic diseases, including animals’ embodied characteristics and behaviours and the relationships between bodies and environments on different farms; and finally the ways in which animal agency and resistance makes a difference to on-farm interventions aiming to prevent or treat lameness and BVD. The paper concludes by arguing that animals’ capacities, and nonhuman difference, should be taken further into account in future policy and practice interventions in endemic disease in farmed animals.

## 1 Introduction

This paper focuses on the more-than-human relationships involved in farmed nonhuman animal (henceforth ‘animal’) health and welfare (AHAW) issues. We draw on empirical work with farmers and their advisers on farms raising cattle and sheep, in northern England. Adding a novel perspective to previous research which has critically examined notions of care in relation to disease (e.g. Harbers 2010, Holloway *et al*. 2023b), we address issues including interspecies relationships between people and their cattle and sheep in rural spaces, everyday farming practices, and nonhuman embodied agency, capacity and subjectivity in relation to endemic health conditions. Animal agency and subjectivity are regarded as co-produced on farms, along with specific endemic conditions, with people and in relation to farming systems and environments. We thus pay attention to the ‘lively’ nature (Barua, 2016; Collard and Dempsey, 2017) of animals as commodities which make a difference to the enactment of AHAW.

AHAW issues in farming have been the subject of extensive scientific and social scientific research (e.g. Bellet *et al*. 2021, Mahon *et al*. 2021). We focus on two contrasting examples: lameness in cattle and sheep, and bovine viral diarrhoea (BVD) in cattle. Described as ‘production conditions’, they are in part an effect of the farming practices and physical conditions in which animals are kept. They have important economic consequences through reduced productivity (e.g. lower milk yields, fertility or growth rates), and are associated with welfare issues (e.g. Bennett & Ijpelaar 2005). Exploring the on-farm situations in which these two conditions are encountered offers different perspectives on animals’ co-production of diseases, because the aetiology of the diseases is so different. BVD is a contagious viral infection, which causes morbidity and mortality. Calves can be infected in utero, if their mothers are ‘transiently infected’ (TI), becoming ‘persistently infected’ (PI) and incurable. They can pass the infection post partum to other animals which become TI, potentially having symptoms which can affect productivity before recovering, and potentially infecting in utero calves. BVD can be detected by tests, and vaccinated against. Testing can be conducted on all animals but is usually focused on calves to identify and cull, PI animals as early as possible (Shortall & Calo 2021). Lameness, in contrast, is a condition with multiple causes, both infectious and non-infectious. Lameness is an abnormality in mobility (Cutress, 2020), affecting animals’ ability to feed and thus their productivity, as well as causing suffering. Its diverse aetiology means that there is a need to determine the cause before responding. Preventative work is carried out at flock/herd level, while treatment in symptomatic animals involves catching and treating individuals, which can pose a welfare issue as cattle and sheep are social species stressed by being separated from their groups. Treatments can involve foot bathing and hoof trimming, along with prophylactic or therapeutic medication, or culling persistently or badly lame animals, and also focus on adapting the farm environment, for example in changing walking surfaces, removing slurry more frequently, or breeding more resilient animals (e.g. Cutress 2020, Lewis & Green 2020, Tunstall *et al*. 2019, Whay *et al*. 2012). Efforts to prevent and treat lameness and BVD tend to revolve around the application of more-or-less standardised protocols such as the Healthy Feet Programme and Five Point Plan for cattle and sheep lameness respectively (see Best *et al*. 2020, Holloway *et al*. 2023a) and the BVDFree England Scheme (https://bvdfree.org.uk/ [accessed 20.2.23]) which aims to combat BVD. However, differences between farms and farmers can make those efforts problematic. Endemic disease situations (Hinchliffe *et al*. 2016) are geographically, temporally and socially variable. As is widely reported in veterinary science literature (e.g. see Kaler & Green 2013, O’Kane et al. 2017, Prosser et al. 2019 in relation to lameness in sheep, Leach et al 2010a; 2010b and Tunstall et al 2021 on lameness in dairy and beef cattle, and Heffernan et al 2016 on BVD), efforts to address endemic disease are complicated by differences in farm environments and management practices, by farmer characteristics, and by the extent to which advice can be made bespoke. Some research with expert sheep lameness consultants suggests that they tend to depend on reference standard care protocols, and do not see a need to take into account farm differences in treating lameness caused by footrot (Winter & Green 2018) while others (Higgins *et al*. 2013) found that vets, in practice, do tailor their advice on footrot depending on their beliefs about what farmers would actually do).

With the exception of bovine tuberculosis (e.g. Enticott 2008) and some research into BVD (e.g. Shortall & Brown 2021, Shortall & Calo 2021) production conditions such as lameness and BVD have been relatively overlooked by social scientists in favour of more exotic, epidemic and zoonotic conditions. There is also little work addressing animal agency, subjectivity and experience in relation to endemic conditions (with some exceptions, see e.g. Shortall & Brown 2021). This paper addresses this gap by exploring nonhuman agency and subjectivity in relation to endemic conditions. This allows us to view diseases as co-produced and multiple realities emerging in situated relationships between people and animals, involving animals’ experiences and behaviours. Co-production here refers to the particular ontological ‘realities’ of these diseases, as described by Mol (2003). Mol uses the example of atherosclerosis in humans to explore how different medical specialisms produce different realities of the condition, varying according to the material-semiotic construction of the disease practised by different doctors. In our case, endemic livestock diseases are fabricated from combinations of the bodies, agencies and experiences of animals alongside those of humans, in specific circumstances. We thus focus on the capacities of animals to co-produce AHAW outcomes, on their experiences, and their ability to comply with or resist farming practices associated with improving AHAW. We explore how animals’ bodies, agency and subjectivities are entangled with specific farmed environments, and on how these entanglements are associated with susceptibilities and/or resiliences to endemic conditions. We ask what difference animals make to constructing the realities of endemic conditions on farms, and to how they are encountered, understood and responded to by the people involved.

First, we review literatures on endemic conditions and concerns for farmed animal welfare; on animal geographies; and on the problematics of care in livestock farming. We then outline our methods before drawing out three inter-related empirical themes concerning how people and animals are together involved in the enactment of endemic conditions. We conclude by arguing that animals’ capacities, agency and subjectivity make a difference to the enactment of interspecies ‘disease situations’ involving people and their cattle and sheep (Hinchliffe *et al*. 2016), and that the variations in human-animal and animal-environment relations which are associated with nonhuman agency, subjectivity and resistance need consideration in further attempts to reduce the prevalence of endemic conditions.

## 2 Endemic conditions, animal geographies, and care

Addressing endemic conditions in farming is complex (Best *et al*. 2020, Holloway *et al*. 2023a, Horseman *et al*. 2014, Shortall & Calo 2021, Yarnall & Thrusfield 2017). Because of their persistence, they have become somewhat normalised within farming, and can remain relatively unnoticed (Wynands *et al*. 2021). Vets have increasingly embraced preventative approaches alongside reactive ‘firefighting’ responses. This implies whole herd/flock health planning which takes a more holistic view of what produces good health outcomes on farms (Bell *et al*. 2006, Clarke & Jones 2011, Kaler & Green 2013), reflecting a longer history of attempts to introduce ‘preventive’ approaches (Woods 2011). Critique more widely has focused on how responses to production conditions focus on treating symptoms or on breeding more resilient animals (see e.g. Nielsen *et al*. 2011), rather than on how these conditions may be effects of particular farming practices or systems (Bellet *et al*. 2021, Holloway & Bear 2021). This means excluding critical questions about ‘industrialised’ livestock farming and the embedded histories of specific human-animal relationships which have produced AHAW problems (Giraud 2019, Holloway *et al*. 2023b). Concern for farm animals is also expressed through the concept of welfare (Buller & Morris 2003, Buller & Roe 2018). While ‘welfarism’ is criticised for legitimising problematic farming practices (Cole 2011), it draws attention to the relational achievement of care (Buller & Roe 2018), acknowledging an intersubjectivity which necessitates thinking about animals’ potential for suffering. To expand on these ideas and apply them to lameness and BVD, we specifically draw on ideas from literature on animal geographies (see e.g. Urbanik 2012) and concepts of care.

Animal geographies pays attention to animal embodiment, subjectivity and agency in different places and in different human-animal relationships (see e.g. Gorman 2017, MacKay 2021, McFarland & Hediger 2009, Pandian 2008). As MacKay writes, ‘animal geographers define animal subjectivity as the ability of animals to live and experience the world as thinking, feeling, sentient and self-conscious beings’; and subjectivity is considered alongside animal agency, where animals ‘exert power in their relations with others, shaping their lives and the lives of (human and nonhuman) others in the process’ (2021: 117). Acknowledging farm animal sentience and subjectivity is thus important (Buller & Roe 2018). This subjectivity is not inherent to animals, but is produced through their relationships with people and farming systems and is thus variable (Gillespie 2017, Holloway 2007, Miele 2016). Key to a geographical perspective is thinking about human-animal relationships in specific places and situations(Holloway & Bear 2021). Research has focused on, for example, the co-constitution of farming places by humans and animals (Gray 1998), the negotiation of situated ethics pertaining to human-animal relationships on farms (Holloway 2001, Holloway 2002, Wilkie 2005, Wilkie 2010), the ‘responsible anthropomorphism’ fostering people’s empathy for animals associated with ‘attending more closely to the understandings of nonhumans garnered from the practice and experience of co-relationality’ (Johnston 2008), and the implications of technological changes in farming for animals’ bodies and experiences and their relationships with people (Holloway 2007, Holloway & Morris 2008).

Wider debates about relationships of care between humans and the nonhuman world are important (Puig de la Bellacasa 2017). As Lawson (2007) argues, the need for care is produced over time, as part of particular social and institutional relationships. While she writes about care for humans, we suggest that farming’s institutional and social systems similarly produce the need for care for animals. This care is direct, embodied and responsive, and enmeshed in legislative frameworks related to, for example, animal welfare and food safety (Buller & Roe 2018). Practices of care have been increasingly problematised in literatures inspired by both feminist and more-than-human thinking (Giraud 2019, Holloway *et al*. 2023b, Puig de la Bellacasa 2017). Here, care is de-sentimentalised and associated with obligation, responsibility and, frequently, hard physical and emotional work (Thomas 1993, Tronto 2020).

The idea of ‘response-ability’ (following Haraway 2008) in relation to another’s needs (see also Brown & Dilley 2012), stems from this literature. Becoming ‘response-able’ means acknowledging that the ‘use’ of nonhuman others affects them, and being able to learn to respond appropriately. We thus ask how farmers become response-able with their animals, in relation to endemic conditions. We focus on care which is necessarily ambiguous (because it relates to animals raised for food production), and co-produced by farmers and their advisers (Holloway *et al*. 2023b). The ambiguities of this kind of care are discussed by several authors (e.g. Gibbs 2021, Harbers 2010, Sneegas 2021), acknowledging how animal sentience does not preclude exploitation and can sustain it (e.g. Arcari *et al*. 2021, Giraud 2019, Williams 2004). This is exemplified in the work of Grandin, who has influenced animal handling techniques based on her understanding of their sentience (Grandin 2019). Another dimension to this is discussed by Gibbs (2021, see also Franklin and Schuurman, 2019) who discusses practices of care for ‘unsound bodies’ (p.375) and how they require a negotiation of what illness or infirmity means within human-animal relationships. While they discuss horses, thinking about cows and sheep affected by endemic conditions as similarly ‘unsound’ contributes to a sense of needing to engage with the embodied realities and effects of infirmity in farmed animals.

We extend these perspectives, centring the animals, exploring the contributions made to a co-production of care by animals themselves, paying attention to their embodied capacities and qualities, and to their subjectivities and agency (for other examples see Bassi *et al*. 2019, Bock *et al*. 2007). This approach also extends work which argues that care for other humans is part of *social* two-way relationships (Rummery & Fine 2012) and is a collaborative practice (Tronto 2020). We argue that animals are similarly involved in collaborative, social relationships, which produce care in specific circumstances. Cole (2011), for example, argues that farming animals forces them into relations of care with humans, and that as such animals are necessarily involved in ‘telling’ people about their welfare needs. Yet at the same time it is important to bear in mind that relations of care in farming might be disrupted by expressions of animal agency and subjectivity, and by the recalcitrance of animal bodies. While Giraud (2019) emphasises how histories of domestication have produced docile animals who do not disrupt (and may facilitate) exploitation, others have emphasised the capacity of animals to resist (Carter & Charles 2013, Gillespie 2016) or to perform ‘divergent conduct’ (Bear & Holloway 2019). Resistance to, or divergence from, the conduct wanted or expected by humans thus becomes an important part of the fabrication of care around farmed animals, because of the ways it affects the performance of care and potential outcomes in terms of welfare, health and productivity.

## 3 Research Methods

We draw on in-depth empirical research with farmers in northern England who raise cattle and/or sheep, and with their advisers, including vets, hoof trimmers and specialist consultants. Research was approved by the University of Hull Faculty of Science and Engineering Research Ethics Committee.

We conducted 29 interviews with farmers with cattle (dairy or beef) and/or sheep enterprises between September 2019 and March 2021 (Table 1). They were selected to illustrate a range of farm types and environments, and included larger and smaller farms, in upland and lowland locations. Research was affected by UK Covid-19 restrictions. Eleven farmer interviews were conducted on-farm, and included observational fieldwork (farm walks, observing the farm spaces and human-animal interactions). Later interviews were conducted online or by telephone, without observation of on-farm care practices. Farmer interviews covered their role and farming biography, the geography and layout of the farm, their approach to and ‘philosophy’ of farming, choice of breeds and breeding practices, their understandings, knowledge and experience of lameness and BVD (in a wider context of thinking about AHAW), and how they worked alone and with others to address these conditions.

We interviewed 21 advisers, between March 2020 and March 2021; all except two remotely. These interviews focused on interviewees’ role and biography, along with discussions of the farms and farmers they worked with, their understandings of and specialist knowledge-practices relating to lameness and/or BVD, their approach to their work and relationships with farmers and animals, and how their work was situated in wider regulatory, policy and research frameworks.

Interviews were recorded, transcribed, and coded with Nvivo software, using a codebook iteratively developed to assist analysis of this dataset (DeCuir-Gunby *et al*. 2011). All authors were involved in codebook development, which was structured around a deductive framework of themes derived from project questions, and subsequently supplemented by inductive codes derived from readings of transcripts. Each interview was coded by two of the authors, using the codebook to ensure consistency.

## 4 Enacting endemic disease with cattle and sheep: subjectivity, agency and embodiment

The following subsections explore how farmed animals’ bodies, agency, subjectivities and capacities are involved in co-producing particular realities of endemic conditions on farms. First, we discuss inter-subjective human-animal relationships. Second, we consider nonhuman components of the ‘disease situations’ associated with endemic conditions. This concept implies that it is something about farming conditions and practices which produce a disease risk (Hinchliffe *et al*. 2016). We develop this here to consider animals’ embodied characteristics and behaviours and the relationships between bodies and environments. Finally, we focus on how animals’ agency and subjectivities affect interventions in response to endemic conditions. We illustrate these themes with comments from interview transcripts. In doing this we do not attempt to show where interviewees are ‘correct’ or ‘incorrect’ according to veterinary science. We are interested in interviewees’ perceptions and practices, which in some cases differ from scientific views of what is correct in ways which can complicate the application of ‘best practice’ on real farms (see Holloway *et al*. 2023a). Our perspective here is that farmers’ perceptions and practices, ‘right’ or ‘wrong’, contribute to the co-production of endemic conditions with their animals.

### Intersubjective relations and responses to endemic disease

Intersubjective relations between people and animals involved the ways animals ‘communicated’ about their experience of endemic conditions through their bodies and behaviours. They also involved opportunities, or obligations, for people to become ‘response-able’ with their animals. As one farmer put it, ‘… they are sentient beings and they do express emotions and I’m sure have feelings and we know that’ (F18). We outline several dimensions to the recognition of this intersubjectivity.

At a fundamental level there was an acknowledgement for many interviewees that animals’ experience of endemic conditions would involve suffering. One vet described lameness in technical terms, then drew in concepts of pain and its effects on animals’ experience;

‘… lameness is musculoskeletal compromise in cattle associated with pain, with pathology and lesions, with affected mobility, compromise to welfare, and potentially therefore compromise to other associated functions like production facility because of ability to express behaviour. So, I think it’s all of those, lameness is a complex of pain, welfare, ability to actually move around and express behaviour’ (A8).

For farmers, this understanding of lameness was associated with noticing how the experience of pain affected animals’ behaviour, enabling them to respond. One explained how they identified cases of lameness by describing a specific instance when;

‘… we went to feed the animals all the rest came forward to the trough and [one bullock] was standing at the back of the yard holding its foot up. Clearly, it’d lost its appetite or lost its ability to move around’ (F9)

The level of intersubjective relationship was extended where farmers recognised the individuality of specific animals, and noticed and responded to individual needs. For example, farmers described how they could identify lameness because they were familiar with individual animals, and felt a sense of shared experience through drawing analogies between human and animal suffering. Discussing lameness in cows, a farmer said;

‘She might literally just have just an ever such a slight hobble or a slight difference in gait but then you know it because you know who she is. You might have a stone in your shoe, you’re going to walk differently, aren’t you and your best friend might go, is there something wrong with you, have you hurt your foot? Oh, no I’ve got a stone in my shoe, it’s as simple as that but your good friend will know because she sees you all the time.’ (F17)

In relation to cows’ experience of BVD, another farmer described their intersubjective encounters with animals, describing a shared experience of illness.

‘… it might look poor or it might look a bit fed up with itself, stood in a corner. You can tell with an animal’s face when they’re ill, just like us. If you’re not having a good day, you’re like [sighs]. An animal is exactly the same. If you know your animals and you’re walking past them, you can tell if they’re not bright in their eye or if their ears are a little bit lax or if they’re just stood there … they’re not feeling well. That’s what you see when an animal has encountered BVD … So it’s like getting a bad cold’ (F3).

In these comments there is a sense of identifying and empathising with cows, and in these cases attempts by interviewees to put themselves in the position of the cow, which is part of the co-production of lameness. While this might prompt concerns about anthropomorphism, and criticism that humans cannot really know that animals’ experience is akin to humans’, we argue that the kind of ‘responsible anthropomorphism’ (Johnston 2008) evidenced by such discussion is away in which people can acknowledge the subjective experience of specific animals. Echoing the empathetic comment above, another farmer said that ‘Generally [lameness results from] when they stand on something hard – it’s like us, if we stand on a bit of Lego it hurts and it’s the same for them really’ (F1). This assumes a shared experience based on similarities between humans and animals that can drive people to respond sympathetically to animals’ pain by making interventions to reduce suffering.

Building on this sense of shared subjective experience, in several interviews there was discussion around the appropriate way for people to behave on farms, which acknowledged that they were dealing with animals who would experience and respond to human behaviour as sentient subjects. This intersubjective encounter would, then, make a difference to people’s ability to detect and respond to endemic disease. As one foot trimmer^1^ said, describing their conduct on-farm;

‘handling the cows properly, handling them in a quiet and considerate manner … You don’t want to be, you know, shouting and doing things like that to the cows. So if you handle them in a quiet and considerate manner … the farmers are very aware of how you handle their cows’ (A2)

For this trimmer, conducting themselves well with cows produces a better outcome in terms of their ability to deliver foot care. In a similar way, a farmer described their intersubjective encounters with their animals;

‘… you’ve got to have a connection with them … you can anticipate their behaviour, you know what they should do, you know what they shouldn’t do, if you can pre-empt how they’re going to react to a situation you know how to behave. Some people aren’t used to cattle will maybe make more noise than is appropriate or move more suddenly than is appropriate or not be in the right place to stop them going through a gap if you’re moving them. That’s what I think of stockmanship, it’s just having that instinct to know how they’re thinking and what they’re going to do’ (F18).

As with the foot trimmer, intersubjectivity is here in part instrumentalised, in connection with the need to move and work with animals. In other interviews, however, the intersubjectivity of human-animal relationships was discussed in ways that emphasised a more sensual dimension to the encounters with nonhuman others.

‘The thing I like about my cows is that they’re all quiet and as well as knowing every one more or less individually, you can walk in amongst them and they don’t bat an eyelid, you can touch them, you can stroke them, they’re used to you and you’re used to them. If anyone strange went in amongst them they would behave differently …’ (F9).

In this comment not only is the embodied, sensual experience of being with cows described, but the cows are ascribed with agency, discriminating between different people and contributing to intersubjective relations.

Discussion of intersubjective relationships involved other ways in which people ascribed ethical value to their animals, and empathised with them, acknowledging their subjective experience. One farmer emphasised the ethical perspective they had developed in relation to their animals, ‘I saw them in their own right rather than seeing them as a financial return’ (F27). While saying that the animals were commercially productive, this farmer valued them as more than just production units, and this meant taking a particular moral perspective on diseases which affected their cattle. As they said in relation to BVD, ‘Well any disease is a waste isn’t it? You try to keep your animals healthy. It’s unfortunate and a shame as well. It’s a shame for the animals concerned’ (F27). This sentiment was echoed by another farmer in saying that,

‘… the last thing you want is [sheep] in pain. An animal in pain is not going to grow. It’s not going to feed a lamb if it’s got a lamb with it. It’s not going to produce milk. Actually, it’s not going to be able to get from A to B without being in pain … Why would you have an animal in pain? You wouldn’t. You wouldn’t do that to it’ (F3)

The entanglement of commercial considerations with a sense of animals’ subjective experience of pain suggests a complex, situated ethical perspective in which an acknowledgement of suffering by sentient nonhuman subjects is important in part because of its effects on agricultural productivity, alongside an ethical sensibility addressing pain as an ethically ‘bad’ phenomenon.

### Producing disease situations

This section considers how animals’ bodies, agency and subjectivity are part of the co-production of disease situations. Key here is a sense of animals’ capacity, combining their bodily attributes and agency. This combination of embodied and agential characteristics makes animals, according to interviewees, more or less susceptible to endemic conditions. Animals’ capacity is intertwined with the physical qualities of the environments they encounter, making disease situations a co-production involving the environment (inside or outside of farm buildings) and the animals themselves.

One farmer articulated this body-environment relationship in describing how their beef suckler cows ‘balanced themselves’, being able to thrive in challenging upland conditions because they embodied particular qualities and exhibited particular behaviours. As they said,

‘They are not bothered about weather, about rough grazing. They are not bothered about climbing over rocks. They’re quite agile. They’re black hoofed. They’re very good on their feet … they prefer to balance themselves so they’re quite a natural breed if that makes sense.’ (F3)

The same farmer kept sheep, and described them in a similar way (pointing to places on a farm map);

‘So if the sheep come into those areas or those areas then if we are going to get lameness … on the rest of the ground they can balance themselves as well and they’re walking on rougher terrain and it just sorts them out somehow’ (F3)

The farmer attributes a lack of susceptibility to lameness in their animals to specific embodied qualities (e.g. black hooves are associated with resilience) and capacities (such as agility and an ability to ‘balance’ themselves as a ‘natural’ breed with good conformation), and a relationship to the environments experienced in some parts of their farm. In this case, the harsher environment of some areas counterintuitively might produce better health outcomes. These animals are seen as belonging in particular environments and when sheep are brought to lower ground for mating and lambing, lameness was more likely to occur.

Other examples focused on animals’ behaviours and how they might be related to environmental conditions which could be managed by the farmer. One farmer (F27) described managing the farm environment to reduce lameness;

‘… if they wander along the same path then they are more likely to transmit [infection] if they’re affected …If the grass is long, they tend to form a track in it as well. So although there’s advantages in having long grass, I have less worms. The sheep tend to form a track in it and therefore they transmit the lameness’.

In such comments, animal agency is acknowledged as important in the disease situation co-producing or mitigating against lameness: the farmer can manage grass length, but the sheep themselves make a crucial difference by (in this case) creating paths and following each other, making infectious lameness more likely. The complexity of the situation is also noted, as according to this farmer keeping the grass short, while reducing lameness, can increase incidence of intestinal worm infections. In another example, a farmer described micro-geographical differences on their farm, between body-environment relationships affecting their sheep and cows. As they described it;

‘we have some land down the road that we took on last year, it’s a lot worse, a lot worse and a lot drier, I can’t work it out, it’s just sand, sandy land down there. The only thing I can think of is that there are no watercourses down there, everything here drinks out of watercourses … The cattle and sheep are crossing these watercourses all of the time … so they are getting their feet washed … We can fetch lame sheep from down the road back up to here and dress them and they are fine within a few weeks, you dress them down there and they never seem to get better’ (F7).

Here, the presence or absence of lameness is associated with a relationship between animals and environmental qualities such as soil type, and with behaviours such as crossing watercourses. Outcomes of the care offered to lame sheep – dressing their feet – are also variable depending on where the treatment happens.

The previous examples refer to situations where animals are outside. Other farmers referred to situations where animals were inside and different body-environment relations were evident. One farmer described how in many dairy farming systems cows are permanently in buildings;

‘Dairy cows, in the main, to try and make some money, sadly, are in 365 days a year. I mean sadly, when you open the door for some of them they don’t want to go out because they’re getting a 50 litre ration^2^ in front of them, a very comfortable bed and they haven’t got to walk far but they are always in that environment and walking on concrete. Yes, I would think that’s why they get [lameness] so badly and they’re under a lot of stress’ (F29).

This description, where a relationship between a stressed body and concrete leads to lameness, contrasts with other dairy farming situations where different cow-environment relationships are evident and which, it is argued, result in lower incidences of lameness. A different farmer described how they bred dairy cows to have resilient bodies with ‘hardy feet’ as, on their farm, the cows needed to walk to grazing, on high altitude, sloping land. At the same time they explained why they bred relatively small cows, in comparison to high-yielding but larger Holstein cows.

‘… we’re quite a high up farm, we haven’t got the best farm in the world. So we need a hardy sort of a cow that can walk out to grass in the summer, walk down our tracks and our lanes. We’re not just flat, they’ve got to go and work for it […] we’ve got to work with the stall length we’ve got rather than have to alter all the sheds. Our stall lengths are only six foot six, seven foot, whereas you really want eight foot for a Holstein, you need to get enough lunge to get up’ (F7).^3^

Here, the farmer emphasises how their cows have the capacity to cope, in a way which combines embodied and subjective qualities, with a specific outside environment, as well as describing how they are bred to fit the restrictions imposed by particular indoor conditions (i.e., small stalls). Both considerations are related to reducing lameness in situations where animals encounter indoor and outdoor environments. This further illustrates how animals and farmed environments are co-produced in relation to, *inter alia*, endemic conditions. Thus, there is a history to disease situations which results in particular animals being present in and created for particular farmed environments, as well as particular economic and other circumstances (Giraud 2019). The importance of breeding histories, and the qualities brought by specific breeds to disease situations, was noted by several farmers. In the first example mentioned in this section, for instance, the cattle breed was described as suited to the farm’s upland environment. Other farmers described how cattle and sheep breeds were co-produced with farm environments and were associated with low levels of lameness in part because of the agency expressed in terms of their mobility as they moved through their environments. A farmer discussed their sheep breed, saying ‘they’ve got such hard feet, they simply do not get footrot^4^ ever […] they’re just incredible sheep and they’re so hardy and they just quietly get on with it. They’re clever, they’re not stupid sheep…’ (F11). In contrast, other farmers referred to bodily conformations and breeds that were associated with lameness. One said that,

‘If you’ve got a sheep that flips its legs like that when it walks, its conformation is all over the place, then how is it going to put equal pressure on its hooves to wear them down naturally because that’s ultimately what happens. They’re treating their feet all the time they’re walking around’ (F3).

This emphasises both the importance of embodied qualities, and the sheep’s agency in walking as a process acting against lameness.

Animals’ embodied and behavioural responses to changes in environment or to new environments were also part of emergent on-farm disease situations. A hoof trimmer discussed the arrival of cows in a new setting;

‘And you’ve got cows maybe … again, because they are in a new environment, they maybe spend a bit too long standing, so that then leads on to bruising [of feet], which then leads on to ulcers. So you’ve got to try and nurse the farm and nurse the cows through that … they just don’t respond well to that new environment’ (A2)

This draws attention to how animals’ subjective experiences are important, but also implies the co-constitution of the farm environment and the animals’ bodies, agency and experience by using the terminology of ‘nursing’ to describe the application of care to both the farm setting and the cows. There is an emphasis on needing to care for the whole farm as ongoing processes imbricating the lively nature of the cows with an evolving farm environment and disease situation.

### Co-producing interventions in endemic disease

This final empirical section discusses the relationships between animal embodiment, agency and subjectivity and interventions made by farmers and advisers in response to lameness and BVD. We examine the difference made by animals as actors in these interventions, and draw attention to the ways in which nonhuman resistance matters in these situations where realities of lameness and BVD are co-produced.

First, animals’ behaviours have to be accounted for in making decisions about interventions such as BVD testing and vaccination regimes. As a vet described it, despite the reduction in infection risk produced by walls and fences designed to stop direct contact between neighbouring herds, ‘nose-to-nose [contact] would still be possible … we really, really push [farmers] to vaccinate because we can’t be sure of their biosecurity’ (A7). In this case, the agency of cows seeking to overcome barriers in their urge to establish physical contact with conspecifics drives the need for vaccination as a biosecurity intervention.

Other examples extend the concern with animal agency to take into account how acknowledgement of animals’ subjective experience makes a difference in planning interventions. A farmer described the implications for their cows of experiencing routine foot trimming procedures. In this intervention, cows are removed from their normal environment, and isolated in a pen for treatment.

‘You are stressing the animal. You are taking her away from her cohort, her 100 cows where she knows her pecking order … They don’t enjoy it. You can make it as attractive as possible for them but they don’t enjoy being pulled away from their mates … You’ve interrupted their natural routine during the day. They’ve come out of the [milking] parlour or, *I go in, I have a bite to eat, I then go down and I cud for hours, then I get up* … Why put that on the cow?’ (F2).

It is worth noting how the farmer temporarily adopts the subject position of the cow (emphasised in italics) as a way of articulating their empathy with bovine experience. A foot trimmer similarly adopted the subject position of cows he described as not being regularly handled;

‘the cows are very, very wild, and quite stressed out, and it’s probably because they are not used to being handled, and they are not used to seeing people. The only time they will see people is when *somebody is going to stick a needle in me or do something bad to me*, so they don’t associate people with nice things’ (A3).

This is a kind of responsible anthropomorphism in which the experience of the cow is assumed to be similar enough to that of the human to make it comprehensible and to matter. The difference this makes was explained by a sheep farmer, arguing that they would refrain from intervention because of the disruption and stress caused to their animals.

‘I can’t think of any circumstances in which we would separate [a sheep] from the flock with the intention of treating them […] they get so freaked out that, you know, the stress isn’t worth it […] The stress of separation is a worse welfare issue than the feet. No, we just foot bath the whole flock and we foot bath more regularly. Our response to lameness is foot bathing the whole flock …’ (F21).

This comment suggests that in some cases, the intervention deployed (treating a whole flock instead of an individual sheep) is in part determined by the acknowledgement of animals’ subjective experience. Here, the sense that the stress caused by separation and individual treatment produces poor welfare outcomes drives an alternative intervention. Drawing attention to the difference that working with different species of animal makes, the same farmer described their process of deciding whether to isolate and inspect cows suspected to be lame;

‘You’ve basically got to take a view, do I bring them in or not? That’s the big decision, do I bring them into the building and keep them in … so you stop them walking around so much, make life easier for them. But it’s a bit stressful, you know, they miss their mates and you know, investigate, is [the lameness] an infection or an injury’ (F21).

Similar examples relate to BVD. One farmer said, ‘What I don’t like, and the cows don’t like it, the needles and the jabbing and the blood samples that we have to take …’ (F18), describing the process needed for BVD testing. This farmer expressed a sense of a shared dislike of a process which affects their willingness to participate in an intervention regarded as key to eradicating BVD.

Some interventions were discussed as affected by the relationships farmers have with specific groups of animals. One farmer described the subjectification of animals, discussing this in terms of what cows learn through their relationships with people and how this might make them more or less risky to handle during interventions such as the one described here in field notes, where a cow might be very protective of her calf;

‘The farmer mentioned he trusts these newer cattle a lot less than the ones he raised himself. The newer cattle to the farm originally came from a holding in which they spent the majority of the year outside with very little human contact [and] they are still flighty. The farmer thinks that what the cattle “learn” as young animals cannot be unlearnt. This is especially the case for the breeding females when the farmer has to ear tag the calves – he feels a lot safer tagging the offspring of the cows he reared himself. Discusses this in terms of trust – both the farmer’s trust in and understanding of the personalities and behaviours of the animal and the cattle’s trust in the farmer’ (F9, farm walk observation notes).

Interventions such as ear tagging, during which tissue samples for BVD testing might also be collected, were differentiated by the nature of the relationships between particular people and particular animals, and the histories and ongoing ‘becoming’ of those relationships, meaning that they were not necessarily implemented consistently, as might be assumed by (for example) vets.

Other examples focused on incidences of resistance, or divergent conduct. Phenomena of resistance and divergence could be produced in relation to the experience of specific moments of intervention. A vet described the embodied struggle associated with inspecting cases of lameness; ‘This is another thing with cattle, the turnover crush, rather than scrapping with ropes and getting kicked in the face and stuff, you can actually restrain the animal properly and have a look’ (A10). Descriptions such as this, where animals’ conduct made treatment difficult and potentially dangerous, produced responses where equipment was used to reduce the capacity to resist, and as such to minimise the potential for them to affect how interventions were made.

Other situations included examples where descriptions of divergent conduct related to animals’ reluctance to perform certain behaviours, and to evade interventions. One farmer explained attempts to train sheep to stand on a chemical pad, as a way of treating lameness.

‘I tried once or twice in the shed to put one of those pads down near the water trough with zinc sulphate in it and they would stand there for a while but that didn’t work so well because you had to train them to go through the gate to drink the water … It took far too long to train them so it didn’t really work very well’ (F27)

A similar example of resistance to treatment was described by a vet who said, about a farm they worked on, that ‘the cows aren’t managing the system where they can be foot bathed particularly easily. They don’t want to go through the parlour and have their feet inspected’ (A7). The cows’ reluctance to take part in lameness treatment further exemplifies the importance of their agency to the ability (or otherwise) of people to intervene in particular ways. Another farmer described this in relation to their sheep, saying that ‘with foot bathing they are able to hop through with three legs. So again it is that system of holding them in it and pushing them in even though they don’t want to’ (F1).

While these acts of divergent conduct could be frustrating and limit what interventions could be performed, other farmers described the physical dangers of interventions alongside the embodied capacities of animals to act against interventions. One farmer described their job as dangerous and told of how they had been injured and hospitalised when a cow bolted. Saying that ‘it wasn’t her fault’, he acknowledged the fear and stress experienced by animals during treatment (F5, farm walk observation notes). Another example was provided by a farmer who was trying to comply with regulations requiring ear tagging of calves, which would also provide a tissue sample for BVD testing.

‘Their mothers glaring over when you’re doing it, it’s positively dangerous. If you don’t catch the [calf] within 24 hours it’s up and running, you’ll have no chance of getting hold of it whatsoever. So it was a real blooming hassle trying to tag those calves at the appropriate time although legally we have to tag those calves within a month … One or two [calves] did get missed or the mothers were too aggressive to work alongside’ (F8).

Here, animal agency and resistance to intervention acts not only against the farmer in the immediate setting of the human-animal relationship, but restricts the performance of biosecurity practices and the government of animal health and welfare.

Thinking about the potential for animals to resist can make farmers reluctant to make specific BVD interventions. A farmer who argued that they did not have BVD on their farm said, ‘I thought “I’m vaccinating against something I don’t have, so this is pointless”. It’s not the cost of the vaccine, it’s the hassle of putting the cow through a crush to put a needle in it, that’s the bit that drives you mad … it’s three hours of hassle of getting covered in shit and stood on’ (F8). This farmer commented on their reluctance to intervene in cases of lameness, for similar reasons. Compared to their sheep, their cattle were harder to handle, so consequently you tend to wait a little bit and see what happens in the hope that if it’s a stone in the foot or something or bruising that it might get better These instances suggest that interventions might sometimes be halted or deferred in response to the risks associated with the embodied capacity of animals to resist.

### The importance of difference: species and diseases

In our empirical analysis, it was evident in that, first, there was more material relating to cows than to sheep (despite both species being represented fairly evenly in the field research), and second, that there was more material relating to lameness than to BVD. The dominance of material about lameness over BVD illustrates how the differences between these endemic conditions are important. Lameness has a more complex aetiology, is more immediately visible in animals and is thus directly encountered, and can be empathised with in terms of a shared capacity to suffer from sore feet. Although there are protocols for addressing lameness, the variability of the condition means that possible interventions are also more diverse. BVD is regarded instead as an ‘invisible’ condition, identified scientifically through testing rather than directly by farmers (although, as seen, farmers can still sympathise with cows with BVD). Interventions are, as a result, more standardised. Overall, then, there is more room for animal agency and subjectivity to make a difference in relation to lameness than BVD.

The prevalence of bovine rather than ovine examples, in comments where interviewees spoke in ways which suggested empathy or responsible anthropomorphism with animals, suggests that human-cow relations are different to human-sheep relations, again in ways which make a difference to the constitution of and care for disease situations. This might be due to a range of factors including the larger size of cows, the (generally) smaller numbers in which they are encountered, the more regular contact between humans and cows, and their greater individual financial value. Farmers said that;

‘… it’s very hard, it’s very hard to treat sheep as individuals and for all sorts of reasons. Like, there’s f***ing loads of them for a start. They all look the same, whatever anybody says, you can’t tell them apart … so you tend to think of them as a group rather than as an individual. Cattle, I definitely think of as individuals, sheep I definitely think of as a group … so it means you’re treating sheep statistically’ (F21).

‘Very rarely [would I call the vet out] for a sheep, purely economics, it’s £35 a call out or even £36 now. Probably a ten-minute consultation charge on top of that and then after you’ve told them what the problem is anyway and then another £20 for doing something, so you end up with a bill which exceeds the economic value of the animal.’ (F8)

This point was confirmed by a consultant who said;

‘So, because at the moment because of the economics of it that one person might be running four, five, 600, 700 sheep … When you start to probably having to scale up flock size to labour unit, that [time] isn’t available. So, you’ve got to, you generally start to manage it as a group rather than as individuals.’ (A17)

In saying this, the consultant contrasted group management with the time needed to treat sheep as individuals, making that case that this wasn’t possible with the larger flocks that contemporary sheep farming economics demanded. These comments illustrate how the co-production of disease by humans and animals is variable, in part associated with different animals’ different embodied qualities which in turn gives them variable presence and value in the perception of farmers.

## Conclusions

As we noted in the introduction, it is well known that differences in farm environments, farming systems and farmers’ knowledges and practices make a difference to the incidence and treatment of endemic livestock disease. We argue here that another set of factors is related to the animals themselves. This includes variability in human-animal and environment-animal relationships, differentiation within and between animal species, and resultant differences in nonhuman embodied capacities, agency, subjectivity and resistance. (see Best *et al*. 2020, Holloway *et al*. 2023a). This set of factors has been under-explored in social scientific research. We have thus added a novel perspective to the literature on endemic disease in farmed animals by focusing on how animals’ embodied agency, capacity and subjectivity matters in the co-production of endemic disease in specific, complex on-farm disease situations, and in thinking about addressing endemic disease, and AHAW issues, in farming. Farmed animals are subject to, and formed in relation to, breeding strategies which have to balance disease resistance, resilience in the face of demanding production requirements, and suitability to specific farm environments, and to interventions aiming to prevent or treat endemic conditions. In this paper we have focused on how animals are involved in the co-production, with people, of the experience and care of endemic conditions on farms. We have explored inter-subjective relations, the exhibiting of symptoms, behaviours which make endemic conditions more or less likely, the embodiment of characteristics which render animals more or less susceptible to particular endemic conditions in particular environments, and animals’ compliance with or resistance to care.

Inter-species co-production of endemic diseases such as lameness and BVD needs to be acknowledged as contributing to the complexity of control programmes. Further research into, and responses to, endemic disease situations needs to take account of the differences we have emphasised, in fostering effective care for AHAW, and to take account of understandings of animal agency and subjectivity in developing policy and practices which might further reduce the prevalence of endemic disease. For example, in the UK, Defra’s Animal Health and Welfare pathway for England has to date focused on veterinary inspection and potential grants for equipment, as ways of improving AHAW (including, specifically, addressing lameness and BVD). Alongside this, further attention might be paid to developing the skills and knowledges of those who work with animals, in recognition that good, skilled stockpeople are sensitised and responsive to animals’ contributions to lameness/BVD disease situations, and work with them in addressing those diseases. This is important together with developing modes of communication between farmers and advisers which help to articulate, on the one hand, ideas of standardised best practice and protocol-driven intervention with on the other hand, the complexity of specific farms, animals and endemic disease situations. The agency, capacities and subjectivities which different animals, as individuals and groups, bring to co-produced disease situations are important considerations for formulating interventions aiming to eradicate or reduce specific endemic conditions.

## Figures and Tables

**Table 1:** Farmer and Adviser interviewees

Farmer (F) no.	Gender	Farm type, location and size	Adviser (A) no.	Gender	Adviser role
F1	female	L, sheep (BS300+)	A1	female	Pharmaceutical representative
F2	male	L, dairy (DC250-299)	A2	male	Cattle hoof trimmer
F3 and F4	female & male	U, beef (BC100-149),sheep (BS300+)	A3	male	Cattle hoof trimmer
F5	male	L, beef (BC50-99)	A4	female	Veterinary consultant
F6	female	U/L, beef (BC150-199), sheep (BS250-299)	A5	female	Levy board staff member
F7	male	U, beef (BC10-49), sheep (BS10-49), dairy (DC100-149)	A6	female	Livestock nutritionist
F8	male	U, beef (BC10-49), sheep (BS150-199)	A7	female	Vet
F9 and F10	female & male	L, beef (BC10-49), sheep (BS10-49)	A8	male	Vet
F11	female	U/L, beef (BC50-99), sheep (BS300+)	A9	male	Veterinary consultant
F12	male	U, beef (BC10-49), sheep (BS300+)	A10	male	Vet
F13	male	U/L, beef (BC50-99), sheep (100-149)(until recent retirement. Now has BC0-9)	A11	female	Vet
F14	male	U, sheep (BS300+)	A12	female	Vet
F15	male	U/L, dairy (DC300+)	A13	male	Farm consultant
F16	male	U, dairy sheep (BS10-49)	A14	male	Veterinary consultant
F17	male	U, dairy (DC100-149)	A15	male	Livestock auctioneer
F18	male	U, dairy (DC10-49), sheep (BS10-49)	A16	female	Vet
F19	male	U, dairy (DC100-149)	A17	female	Farm consultant
F20	male	U, beef (BC100-149), sheep (BS300+)	A18	female	Farm consultant
F21	male	L, beef (ND), sheep (BS300+)	A19	female	Assurance scheme assessor
F22	male	U, beef (BC10-49), sheep (BS100-149)	A20	male	Vet
F23	male	U, beef (BC10-49), sheep (BS300+)	Adviser Group	2 female & 1 male	Provide services for vets
F24	female	U, beef (BC10-49), sheep (BS200-249)			
F25	male	L, beef (BC250-299)			
F26	female	L, sheep (BS10-49)			
F27	male	U, beef (ND), sheep (ND)			
F28	male	U, sheep (BS300+)			
F29	male	U, beef (BC300+), sheep (BS300+)			

**‘Farm type and size’ column includes an indication of location (U = upland; L = lowland), and of farm size using a categorisation of numbers of breeding cattle and sheep as indicated by farmers in a pre-interview questionnaire, although some farms had large numbers of non-breeding animals as well as other enterprises. BC = breeding beef cattle; DC = dairy cattle; BS = breeding sheep; ND = animals present but data not given**.

## Footnotes

1 Foot trimmers are engaged by farmers to trim excessive hoof growth or to treat causes of lameness in hooves.

2 i.e., the cows are being well fed to increase milk yields.

3 6 feet and 6 inches is approximately 2m, 8 feet = approximately 2.4m. ‘Lunge’ refers to the forward movement cows make when rising; sufficient space is needed for cows to lunge.

4 Footrot is a bacteriological disease causing lameness
